# A Novel Decision Aid Improves Quality of Reproductive Decision-Making and Pregnancy Knowledge for Women with Inflammatory Bowel Disease

**DOI:** 10.1007/s10620-022-07494-9

**Published:** 2022-04-30

**Authors:** Grace Wang, Neda Karimi, Laura Willmann, Joseph Pipicella, Joseph Descallar, Katie O’Connor, Luiza Peculis, Yvette Leung, Susan Connor, Vivian Huang, Astrid-Jane Williams

**Affiliations:** 1 Faculty of Medicine, University of Toronto, 1 King’s College Cir, Toronto, M5S 1A8 Canada; 2 South Western Sydney Clinical School, The University of New South Wales, Goulburn St, Liverpool, NSW 2170 Australia; 3 Ingham Institute for Applied Medical Research, 1 Campbell Street, Liverpool, NSW 2170 Australia; 4 Department of Gastroenterology and Hepatology, Liverpool Hospital, 75 Elizabeth St, Liverpool, NSW 2170 Australia; 5 Department of Gastroenterology, Mount Sinai Hospital, 600 University Ave, Toronto, M5G 1X5 Canada; 6 Department of Maternity and Gynaecology, Liverpool Hospital, 75 Elizabeth St, Liverpool, NSW 2170 Australia; 7 Department of Gastroenterology, University of British Columbia, 2775 Laurel Street, Vancouver, V5Z 1M9 Canada

**Keywords:** Inflammatory bowel disease, Pregnancy, Conception, Decision making, Decision aid

## Abstract

**Background:**

Women with inflammatory bowel disease (IBD) with poor IBD-specific reproductive knowledge experience more childlessness and fear of IBD medications in pregnancy. The Pregnancy in IBD Decision Aid (PIDA), developed by an international multidisciplinary team, offers personalized online decision support regarding pregnancy in IBD.

**Aims:**

Assess the impact of PIDA on quality of reproductive decision-making and pregnancy-related knowledge among preconception (PC) and pregnant patients with IBD, and evaluate acceptability to patients and clinicians.

**Methods:**

PC and pregnant patients with IBD aged 18–45 completed questionnaires pre- and post-PIDA to assess quality of decision-making (Decisional Conflict Scale (DCS); Decision Self-Efficacy Scale (DSES) and IBD-in-pregnancy knowledge (Crohn's and Colitis Pregnancy Knowledge Score (CCPKnow)). Paired t test assessed for differences pre- and post-PIDA. Patients and clinicians completed acceptability surveys.

**Results:**

DCS and DSES were completed by 74 patients (42 Crohn’s disease, 32 ulcerative colitis); 41 PC and 33 pregnant. DCS improved significantly post-PIDA in PC patients regarding pregnancy planning (*t*(40) = 4.83, *p* < 0.0001, Cohen’s *d*_*z*_ = 0.75) and in pregnant patients regarding medication management (*t*(32) = 2.37, *p* = 0.0242, *d*_*z*_ = 0.41). DSES for PC patients improved significantly post-PIDA (*t*(40) = -3.56, *p* = 0.001, *d*_*z*_ = -0.56). CCPKnow improved significantly post-PIDA in PC (*t*(42) = 4.93, *p* < 0.0001, d_z_ = -0.75) and pregnant patients (*t*(32) = 5.1, *p* < 0.0001, *d*_*z*_ = -0.89). PIDA was deemed optimal for length, readability, and content amount and considered highly useful by patients (*n* = 73) and clinicians (*n* = 14).

**Conclusions:**

Patients using PIDA developed an improved quality of reproductive decision-making and IBD-in-pregnancy knowledge. PIDA is an accessible tool that can empower women with IBD to make values-congruent, evidence-based decisions regarding pregnancy and may reduce voluntary childlessness.

**Supplementary Information:**

The online version contains supplementary material available at 10.1007/s10620-022-07494-9.

## Introduction

Inflammatory bowel disease (IBD), including ulcerative colitis (UC) and Crohn’s disease (CD), commonly affects reproductive-aged women. Women with IBD experience more “voluntary childlessness,” at rates of 18% in CD and 14% in UC, compared to 6.2% in the general population [1]. This may be due to misconceptions surrounding IBD therapies in pregnancy [2–4]. Despite robust safety evidence for many IBD medications in pregnancy [5–7], this knowledge remains deficient for many patients and generalist clinicians[8–10]. Only half of surveyed Canadian physicians felt comfortable managing pregnant patients with IBD, which may explain the wide variability in physician practice for this population [9]. Only a third of reproductive-aged women with IBD had discussed family planning with their general practitioner [11]. These knowledge gaps should be addressed, as inappropriate medication cessation during conception is not uncommon [8] and increases risk of disease relapse. Active disease in the antenatal period is associated with low birth weight, premature birth, pregnancy loss, and infant infection [7, 12–14].

As therapeutic complexity increases, patients desire more education on the intersection of IBD and pregnancy[15]. Women with IBD who receive specialized preconception (PC), intrapartum, and post-partum counseling experience enhanced IBD-related pregnancy knowledge and fewer disease flares, thereby translating into improved maternal and fetal outcomes[16, 17]; however, few patients have access to specialized clinics. Websites and information sheets may enhance IBD-specific reproductive knowledge [15, 18], but patients should also be equipped with tools to improve their quality of decision making. The tenets of high-quality patient decision making include (1) recognizing a decision must be made, (2) feeling informed about options and outcomes, (3) being clear about values and preferences, (4) having facilitated discussion of goals with healthcare providers, and (5) being involved in decisions[19]. Decision aids (DAs) present evidence in patient-centric formats and prepare patients for shared decision making (SDM) with their healthcare providers. They are designed to improve knowledge as well as the decision-making process [19]. Women with IBD have voiced a desire for a DA for pregnancy [20].

In order to address this unmet need, we developed the interactive, web-based Pregnancy in IBD Decision Aid (PIDA) following International Patient Decision Aids Standards (IPDAS) [21, 22] with an international steering committee comprised of patient representatives and experts from IBD, general gastroenterology, obstetrics, obstetric medicine, pediatric gastroenterology, perinatal pharmacoepidemiology, SDM, and knowledge translation. Patient and clinician focus groups, a systematic review of the pregnancy-IBD literature, and in-depth iterative reviews of content topics were performed to guide PIDA development. PIDA offers personalized decision support for fertility, pregnancy, and post-partum concerns; it is thus relevant for all reproductive stages. PIDA focuses on two key decisions: (1) possibility and timing of conception for PC patients and (2) medication management in pregnancy for pregnant patients. These decisions are consistent with patient priorities reported in the literature [23].

This study conducted pilot testing of PIDA to assess its impact on the quality of reproductive decision-making and IBD-related pregnancy knowledge among PC and pregnant women with IBD. In addition, we determined PIDA’s acceptability among patients and clinicians.

## Methods

### Study Design

This multi-national study used a pretest–post-test design to assess PIDA’s effectiveness in improving quality of decision-making and pregnancy-related knowledge for reproductive-aged women with IBD. We enrolled female patients aged 18–45 years with IBD who were either PC (considering pregnancy in the future but more than 12 months post-partum if recently pregnant) or pregnant. Patients were recruited from two IBD clinics at (1) Mount Sinai Hospital, Toronto, Canada (affiliated with University of Toronto), and (2) Liverpool Hospital, Sydney, Australia (affiliated with University of New South Wales). Patients were also recruited via social media advertisements through IBD advocacy groups. Consenting patients were given a link to a secure webpage via Research Electronic Data Capture (REDCap) software and asked to complete a demographics questionnaire and pre-intervention assessments. Following baseline assessments, patients viewed PIDA in full, then completed post-intervention assessments within two weeks.

Clinician participants from gastroenterology, obstetrics, maternal–fetal medicine, and primary care were identified through the research teams’ professional networks and invited by email. Consenting clinicians reviewed PIDA and completed the clinician acceptability survey.

### Intervention

The PIDA prototype can be found at http://ibdpregnancyaid.com. PIDA “walks” the user through chapters that can be viewed as often as desired (Appendix 1)[22]. The tool starts with general education around IBD and family planning and then tailors subsequent chapters to the user’s pregnancy status and IBD characteristics. Finally, PIDA provides patients with an individualized summary that can be downloaded and used for reference or for discussion with their providers.

### Outcome Measures

The following outcome measures were used to assess quality of decision making: (1) Decisional Conflict Scale (DCS) [24] (primary outcome) completed pre- and post-PIDA, (2) Decision Self-Efficacy Scale (DSES) [25] completed pre- and post-PIDA, and (3) Preparation for Decision Making (PrepDM) scale [26] completed post-PIDA. DCS measures (1) personal perception of uncertainty in making a health-related decision, (2) factors contributing to the uncertainty, and (3) the extent to which consumers agree that their decision was informed, consistent with personal values, and would be implemented. DSES measures personal belief in one’s ability to make an informed decision. PrepDM assesses a patient’s perception of how useful a DA is in helping them recognize the need to make a decision, appreciate their values in relation to the decision, prepare to communicate with their provider, and make a health-related decision. Together, these validated scales and their sub-scores measure all five constructs that comprise decision-making quality [19].

To measure pregnancy-related knowledge, we used the Crohn’s and Colitis Pregnancy Knowledge Score (CCPKnow) [27] and completed pre- and post-PIDA. CCPKnow assesses knowledge regarding conception, IBD inheritance, medication use (peri-conception, pregnancy, and breastfeeding), congenital abnormalities, and mode of delivery.

PIDA’s acceptability was assessed using a five-point Likert scale designed based on tools from previous decision-aid studies [28–30]. It collected patients’ and clinicians’ views on readability, length, amount of information, and usefulness (Appendix 2, 3), as well as free text comments.

We also measured participation and retention rates. We defined participation rate as the percentage of approached eligible patients who participated in the study. Retention rate was defined as the percentage of patients assessed and analyzed with the primary outcome (DCS).

### Sample Size

Our target sample size was a minimum of 60 patients overall with a minimum of 30 in each of the PC and pregnant groups across both sites. This target was based on general sample size recommendations for pilot studies [31]; further, 80% of usability problems are revealed with 10 participants, increasing to 95% with 20 participants [32].

### Statistical Analysis

Baseline characteristics were summarized using counts and percentages for categorical variables and means and standard deviations for numeric variables. We performed paired t tests between the pre- and post-test scores for DCS (total score and 5 subscores), DSES (total score), and CCPKnow (total score). Cohen’s d_z_, the effect size on the user before and after PIDA, was calculated. The PrepDM subscores were summarized using means and standard deviations. Acceptability of PIDA was analyzed using descriptive and frequency analysis. Nominal symmetry test was used to compare patients’ pregnancy-related decisions before and after reviewing PIDA. Participation and retention rates were calculated using counts and percentages. Analyses were conducted using SAS Enterprise Guide version 8.2 and R version 4.0.3. Statistical significance was set at *p* < 0.05 (two-tailed tests).

## Results

### Participation and Retention Rates

**Fig. 1:**
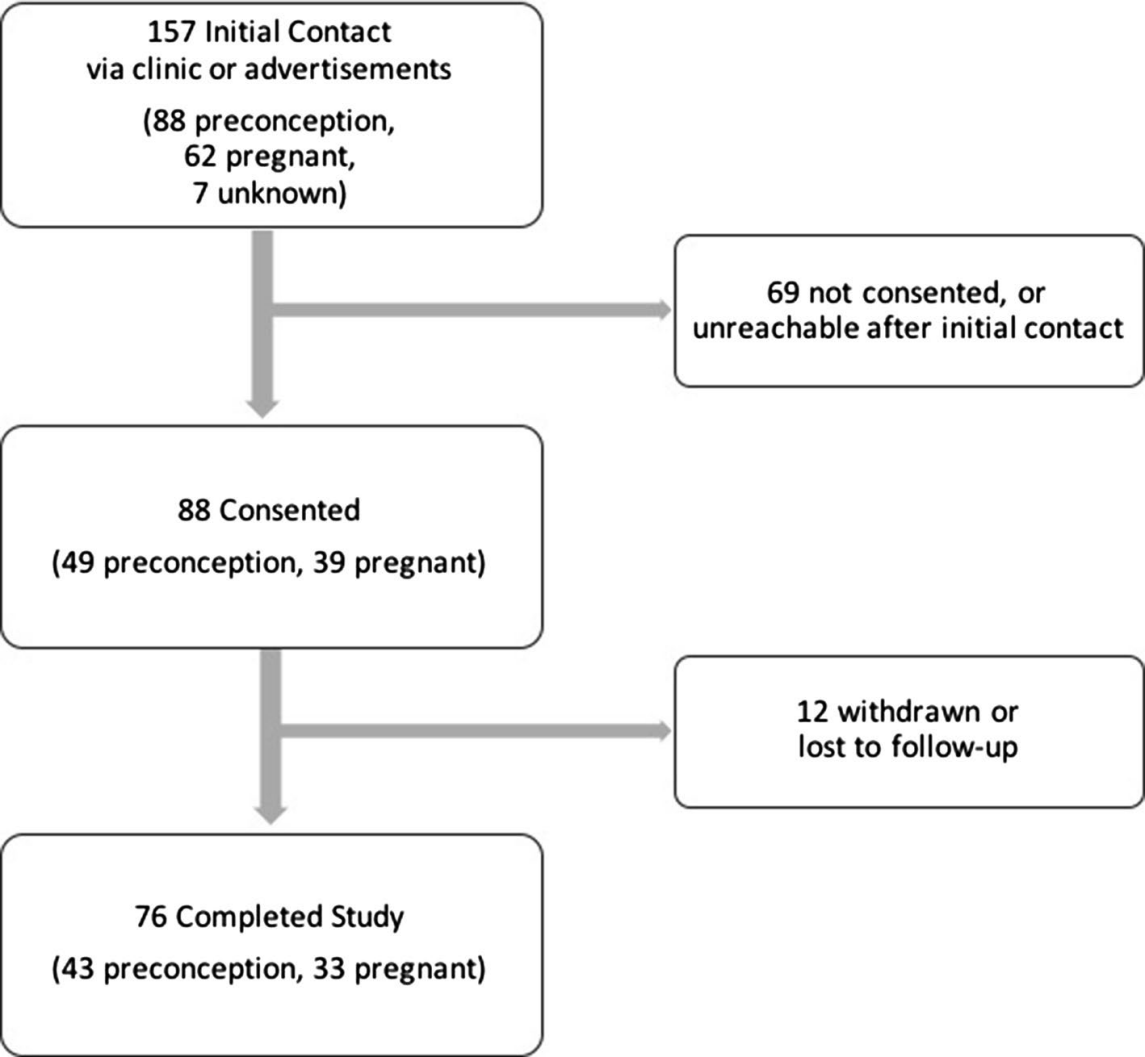
Recruitment and Retention of Patient Participants

One hundred fifty-seven patients responded to recruitment in clinics or advertisements (Fig. 1). Seven were unreachable after initial contact. Of the remaining 88 PC and 62 pregnant patients, 49 PC patients and 39 pregnant patients consented (participation rates: PC: 55.7%, pregnant: 62.9%). Six PC patients and six pregnant patients withdrew or were lost to follow-up. Forty-three PC patients and 33 pregnant patients completed the study (retention rates: PC: 87.8%, pregnant: 84.6%). The details of further missing data for each outcome measure are presented in Online Resource 1.

### Demographics

The study population included a total of 76 patients with IBD (Table 1) and 17 clinicians. Of the patients, 43 (56.6%) were PC and 33 (43.4%) were pregnant, and over half had CD (*n* = 43, 56.6%). Approximately 90% of patients were married or living with a partner (*n* = 68), two-thirds had attained a university degree (undergraduate: *n* = 25, 32.9%; post-graduate: *n* = 26, 34.2%), and 70% had an annual household income of $100,000 or more in their respective currency (*n* = 51).

**Table 1:** Demographics of preconception and pregnant patient participants

		Preconception		Pregnant		Total	
		*n*	%	*n*	%	*n*	%
Diagnosis	Crohn's disease	25	58.14	18	54.55	43	56.58
	Ulcerative colitis	18	41.86	15	45.45	33	43.42
Marital status	Single (never married)	7	16.3	1	3	8	10.53
	Married	27	62.8	29	87.9	56	73.68
	Common law/living with a partner/living as married	9	20.9	3	9.1	12	15.79
Partner diagnosed with IBD	No	42	97.7	32	97	74	97.37
	Yes	1	2.3	1	3	2	2.63
Level of education	Less than high school diploma	2	4.7	1	3	3	3.95
	Completed high school diploma	6	14	2	6.1	8	10.53
	Completed trade, technical, vocational or business school	5	11.6	9	27.3	14	18.42
	Completed university undergraduate degree	14	32.6	11	33.3	25	32.89
	Completed post-graduate degree	16	37.2	10	30.3	26	34.21
Annual Household Income (in respective currency: AUD or CAD)	Less than $20,000	2	4.88	2	6.25	4	5.48
	$20,000—$39,900	1	2.44	1	3.125	2	2.74
	$40,000—$69,900	6	14.63	2	6.25	8	10.96
	$70,000—$99,900	5	12.20	3	9.375	8	10.96
	$100,000 or more	27	65.85	24	75	51	69.86
	Missing	2		1		3	

The 17 clinicians were from Australia, Canada, Denmark, and the Netherlands and included IBD specialists (*n* = 10), general gastroenterologists (*n* = 3), an obstetrician, an IBD nurse, a nurse practitioner, and a general practitioner. Eight clinicians had over ten years and five clinicians had fewer than five years of clinical experience. Nine clinicians (52.9%) had additional training or expertise in pregnancy in IBD.

### Decisional Conflict

Forty-one PC patients and 33 pregnant patients completed the DCS pre- and post-PIDA. Prior to reviewing PIDA, the mean total DCS score (0 = no decisional conflict, 100 = extremely high decisional conflict) was 38.95 (SD 21.31) for PC patients and 23.30 (13.07) for pregnant patients. After reviewing PIDA, the mean total DCS score decreased to 26.77 (16.79) in the PC group (*t*(40) = 4.83, *p* < 0.0001, Cohen’s *d*_*z*_ = 0.75) and 18.99 (17.06) in the pregnant group (*t*(32) = 2.37, *p* = 0.0242, *d*_*z*_ = 0.41), indicating a statistically significant improvement in the DCS score in both groups. Improvement in decisional conflict was more global in the PC group compared to the pregnant group as indicated by a statistically significant decrease in all five subscores for PC patients (Informed, Values clarity, Support, Uncertainty, and Effective decision) compared to a statistically significant decrease in only two subscores for pregnant patients (Informed and Uncertainty) (Online Resource 2 and 3).

### Self-Efficacy

Forty-one PC patients and 33 pregnant patients completed the DSES pre- and post-PIDA. Among PC patients, the mean DSES score (0 = no self-efficacy, 100 = extremely self-efficacious) increased from 78.81 (20.21) to 85.42 (15.64) after reviewing PIDA (*t*(40) = -3.56, *p* = 0.001, *d*_*z*_ = −0.56. The mean DSES score increased for pregnant patients post-PIDA from 82.58 (17.97) to 86.91 (18.59); this change trended toward significance *(t*(32) = −2.01, *p* = 0.0525, *d*_*z*_ = −0.35).

### Preparation for Decision Making

Forty-two PC and 33 pregnant patients completed PrepDM after reviewing PIDA. Both PC and pregnant patients found PIDA useful in helping them make pregnancy-related decisions in the context of IBD (PC: mean = 3.98 (0.76); pregnant: 3.91 (0.74); 1 = not at all, 5 = a great deal). Descriptive statistics of responses to individual PrepDM questions are displayed in Table 2.

**Table 2:** Preparation for decision making scores after completing PIDA, according to reproductive status

	*N*	Question	Mean	SD	Min	Median	Max
Preconception	42	Q1. Help you recognize decision needs to be made	3.60	1.15	1	4	5
		Q2. Prepare you to make a better decision	4.00	1.13	1	4	5
		Q3. Help you think about pros and cons of each option	3.98	1.09	1	4	5
		Q4. Help you think about which pros and cons are most important	3.98	0.84	2	4	5
		Q5. Help you know the decision depends on what matters most to you	4.00	0.94	2	4	5
		Q6. Help you organize your own thoughts about decision	4.10	0.98	1	4	5
		Q7. Help you think about how involved you want to be in decision	3.93	1.07	1	4	5
		Q8. Help you identify questions you want to ask	4.02	1.07	1	4	5
		Q9. Prepare you to talk to your doctor about what matters most	4.19	0.86	2	4	5
Pregnant	33	Q1. Help you recognize decision needs to be made	3.79	1.083	1	4	5
		Q2. Prepare you to make a better decision	3.82	1.074	1	4	5
		Q3. Help you think about pros and cons of each option	3.82	1.103	1	4	5
		Q4. Help you think about which pros and cons are most important	3.79	1.023	1	4	5
		Q5. Help you know that decision depends on what matters most to you	3.94	.933	2	4	5
		Q6. Help you organize your own thoughts about decision	3.85	1.034	1	4	5
		Q7. Help you think about how involved you want to be in decision	4.06	.966	1	4	5
		Q8. Help you identify questions you want to ask	4.03	.951	2	4	5
		Q9. Prepare you to talk to your doctor about what matters most	4.12	.992	2	4	5

### Pregnancy-Related IBD Knowledge

The mean baseline CCPKnow score among PC patients was 9.95 (3.84) out of 18 (“adequate” knowledge level); this increased by 28.7% to 12.81 (3.98) (“good”) after reviewing PIDA. Similarly, for pregnant patients, mean CCPKnow score increased by 20.4% from 10.52 (3.92) (“adequate”) to 12.67 (3.14) (“good”) after reviewing PIDA. These results demonstrate a statistically significant improvement in IBD-related reproductive knowledge in both PC (*t*(42) = 4.93, *p* < 0.0001, *d*_*z*_ = −0.75) and pregnant groups (*t*(32) = 5.1, *p* < 0.0001, *d*_*z*_ = −0.89).

### Pregnancy-Related Decisions in the Context of IBD

Prior to reviewing PIDA, 19 (47.5%) PC patients wanted children but were afraid of IBD-related complications. After reviewing PIDA, eight of those patients (20% of the PC group) reported no major concerns regarding pregnancy in the context of IBD (*p* = 0.00781). Among pregnant patients, no statistically significant changes were observed pre- to post-PIDA in their decision on intrapartum medication management (Online Resource 4 and 5).

### Patient Acceptability of PIDA

Acceptability questionnaires were completed by 42 PC and 33 pregnant patients (Table 3). Both cohorts found PIDA useful for themselves (PC: mean = 4.12 (1.02); pregnant: 4.06 (0.83); 1 = more confusing, 3 = no difference, 5 = useful) and would recommend it to others (PC: 4.00 (1.06); pregnant: 4.00 (1.03); 1 = not at all, 3 = suggest, 5 = highly recommend). Patients found PIDA helpful in identifying their values (PC: 3.52 (1.02); pregnant: 3.79 (0.93); 1 = not at all, 3 = adequate, 5 = very well). PIDA’s length (PC: 3.07 (0.52); pregnant: 3.03 (0.31); 1 = too short, 3 = adequate, 5 = excessive), readability (PC: 3.10 (0.62); pregnant: 3.09 (0.29); 1 = too simplified, 3 = appropriate, 5 = too challenging) and content amount (PC: 2.88 (0.89); pregnant: 2.94 (0.70); 1 = limited, 3 = appropriate, 5 = excessive) were perceived to be appropriate by all patients.

**Table 3:** Acceptability rankings for PIDA in pre-defined domains, according to preconception patients, pregnant patients, and clinician participants

Arm		*n*	Mean	Standard Deviation	Lower 95% CI	Upper 95% CI
Preconception	LENGTH	41	3.07	0.52	2.91	3.24
	READABILITY	42	3.10	0.62	2.90	3.29
	CONTENT AMOUNT	42	2.88	0.89	2.60	3.16
	USEFULNESS (for me)	42	4.12	1.02	3.80	4.44
	USEFULNESS (for others)	42	4.00	1.06	3.67	4.33
	VALUES	42	3.52	1.02	3.21	3.84
Pregnant	LENGTH	32	3.03	0.31	2.92	3.14
	READABILITY	33	3.09	0.29	2.99	3.19
	CONTENT AMOUNT	33	2.94	0.70	2.69	3.19
	USEFULNESS (for me)	33	4.06	0.83	3.77	4.35
	USEFULNESS (for others)	33	4.00	1.03	3.63	4.37
	VALUES	33	3.79	0.93	3.46	4.12
Clinician	LENGTH	17	3.24	0.56	3.00	3.00
	READABILITY	17	3.24	0.75	3.00	3.00
	CONTENT AMOUNT	17	3.35	0.70	3.00	3.50
	USEFULNESS (for me)	17	4.82	0.73	5.00	5.00
	USEFULNESS (for others)	17	4.71	0.77	5.00	5.00
	VALUES	17	4.29	0.99	4.00	5.00

### Clinician Acceptability of PIDA

Clinicians scored PIDA’s length (*M* = 3.24 (0.56)), readability (*M* = 3.24 (0.75)), and content amount (*M* = 3.35 (0.70)) as appropriate (1 = too simplified, 3 = appropriate, 5 = too challenging; Table 3). They reported that PIDA was a useful tool for them (M = 4.82 (0.73)) and would recommend it to patients (*M* = 4.71 (0.77)). They found PIDA helpful in identifying patients’ values (*M* = 4.29 (0.99); 1 = not at all, 3 = adequate, 5 = very well). Clinicians also qualitatively shared their views about PIDA; this feedback is summarized in Online Resource 6.

## Discussion

The quality of decision making in PC and pregnant patients with IBD significantly improved after reviewing our novel decision aid, PIDA. High-quality decision making involves minimal decisional conflict, high perceived self-efficacy in the process, and consideration of one’s values. After using PIDA, decisional conflict, measured by the DCS, decreased significantly from 38.95 to 26.77 for PC patients (large effect size) and from 23.30 to 18.99 for pregnant patients (medium effect size). DCS scores below 25 are associated with implementing decisions; scores above 37.5 are associated with decision delay or feeling unsure about implementation [24]. Improvement in decisional conflict was more comprehensive in the PC group, as indicated by a significant decrease in all five subscores for PC patients compared to two subscores for pregnant patients. Notably, decisional conflict is an independent predictor of propensity to ascribe fault to physicians; in an Australian study, for every unit increase in DCS, patients were 19% more likely to blame their physicians for poor outcomes related to prostate cancer screening decisions [33].

Further, patients developed enhanced self-efficacy and a stronger belief in their ability to make effective pregnancy decisions after using PIDA. The DSES revealed a significant, medium effect size in PC patient, and a small-to-medium but non-significant effect size in pregnant patients. The smaller DSES improvement in pregnant women is plausibly because they had already made several reproductive decisions.

Improving quality of decision making through tools such as PIDA is essential for values-concordant care [34]. Patients value being involved in treatment decisions, and are disappointed by the lack of opportunities to do so [35]. A review of 134 observational studies found that patients without opportunities to participate in health discussions were more likely to be misdiagnosed and undergo potentially harmful, values-discordant treatments [36]*.* Lower patient participation is also tied to treatment non-adherence, which may worsen disease activity [37, 38]. Strategies that facilitate SDM, including DAs, offer patients ownership over disease control and may improve outcomes and reduce healthcare expenditure. For example, self-driven management of stable UC was not only preferred by patients, but also accelerated treatment provision and reduced medical visits without increasing morbidity [39]. A systematic review of 115 trials of DAs demonstrated that they increase knowledge and the likelihood that therapies align with patient values [40]. In a feasibility study of a DA for UC, patients reported that the DA enhanced their understanding of UC therapy and improved their ability to participate in SDM effectively and confidently [41]. Further, patients in our study found PIDA helped them identify their values.

Reproductive decision making is complex as women must consider maternal and neonatal aspects; as such, SDM is instrumental and many DAs exist [42]. Few cater to reproductive-aged women with chronic conditions: A pregnancy-related DA exists for rheumatoid arthritis [43], multiple sclerosis [44], epilepsy [28], depression [45], and breast cancer [46]; until now, no such DA had existed for IBD. Notably, these DAs focused on PC patients contemplating the motherhood decision: the choice to forego, start, or enlarge a family. In contrast, PIDA was designed to cover all reproductive stages: motherhood decisions with PC patients and intrapartum decisions with pregnant patients, while information pertaining to the post-partum stage has also been included.

Patients’ IBD-specific pregnancy knowledge improved after utilizing PIDA. CCPKnow scores increased with a large effect size and an improvement from an “adequate” to “good” level of knowledge for both PC and pregnant patients. Half of PC patients wanted children but were afraid to due to their IBD. After using PIDA, half of these patients no longer had major IBD-related pregnancy concerns. Knowledge deficits surrounding pregnancy and IBD are common [47] and associated with attitudes contrary to medical evidence [3] and ultimately contribute to the higher rates of childlessness observed in women with IBD [2]. For example, unfounded fertility concerns are common in nulliparous women with IBD and may link to childlessness [2, 47]; yet, they only seek fertility advice at the same rate as the general population [23, 48] and may not have these fears dispelled. Reassuringly, fertility counseling significantly increases consideration of pregnancy by patients with IBD [47]. Thus, patient education is invaluable in combating misinformation and improving reproductive knowledge [49–51]. PIDA could reduce childlessness among women who would have otherwise avoided pregnancy due to unfounded fears about their IBD.

If PIDA could improve patient knowledge, it could impact medication adherence. Patients with limited knowledge may believe that IBD therapies broadly are teratogenic [3], which leads to inappropriate medication cessation [8] and higher potential for active disease—the greatest threat to pregnancy outcomes^34^. In one study, half of patients who were pregnant or attempting to conceive had stopped IBD therapy, and half of these patients did so without medical advice [8]. A longitudinal study of 138 patients found that poor medication knowledge led to non-adherence, which led to increased IBD relapses [52]. Fortunately, safety data on IBD therapies in pregnancy are being translated into patient resources [53]. By delivering knowledge to patients in a personalized manner, PIDA may improve medication adherence and optimize disease activity, which may impact obstetric or fetal outcomes. Further studies are needed to evaluate these specific outcomes.

Regarding acceptability, patients deemed PIDA to be appropriate and highly useful, and would recommend it to others. Similarly, clinicians perceived PIDA to be acceptable and highly useful for themselves and patients. Clinician endorsement of DAs is necessary for their ongoing use; fortunately, several benefits exist for providers. In a Cochrane review, DAs enhanced the patient-clinician consultation in nine of ten studies with little to no impact on visit length [40] (it may be completed pre-visit [54]). DAs increase clinician satisfaction [55], as well as patient satisfaction [56] and trust in their physician[57]. Based on such literature, PIDA may strengthen patients’ therapeutic alliance with their physician.

In the future, we plan to further refine PIDA by adding keyword search functions and accessibility features such as audio, video, and expanded language availability. This study will inform the design of a randomized controlled trial, after which we hope to have a validated DA that can support reproductive decision making in IBD. We plan to promote the sustained use of PIDA through patient advocacy groups and integration into electronic medical records [58] and assess its impact on treatment adherence, disease activity, and pregnancy outcomes. We also envision PIDA as a DA prototype to be easily adjusted for use in other chronic conditions.

Our study has several limitations. Firstly, while our sample size was guided a priori by other pilot studies, the number of patients did limit our ability to capture a broader perspective and analyze subgroups. Further, inherent to our recruitment method is the risk of self-selection bias, as those enrolled may possess a particularly poor understanding of, or special interest in, pregnancy and IBD. PIDA’s readability is another area for improvement. In North America and Australia, it is recommended that patient education is written at a grade eight level [59]. PIDA surpassed this given the terminology associated with IBD therapy and reproduction; accordingly, PIDA is designed to be interpreted with clinician guidance for full comprehension and decision making. Nonetheless, PIDA would benefit from the above accessibility features for patients with lower literacy. Another weakness is our overrepresentation of highly educated women of high socioeconomic status. This stems from multiple reasons, including accessibility to tertiary care, health literacy, readability level, and technological resources or time to complete uncompensated surveys. Thus, it is unclear whether PIDA would generate a similarly favorable response in the general population. Reassuringly, DAs do improve outcomes for disadvantaged patients and may in fact benefit such groups more than those with higher socioeconomic status [60]. We hope PIDA will help bridge health inequities in patient education.

Overall, our pilot study has shown favorable and compelling results. PIDA is an accessible, individualized intervention that may be widely circulated and fulfills an unmet need in patient decisional support. It can empower women to build IBD- and pregnancy-related knowledge, gain self-efficacy, and make values-congruent, evidence-based decisions about their IBD in the context of family planning. There may be the potential for minimization of voluntary childlessness. While we cannot make firm conclusions about pregnancy-related outcomes, we believe that the insight and decisional support gained from PIDA has the potential to improve disease control and positively impact maternal and fetal outcomes. We look forward to validating PIDA for PC and pregnant patients in an upcoming multi-national randomized controlled trial.

### Electronic supplementary material

Below is the link to the electronic supplementary material.

Supplementary file1 (DOCX 34 KB)

## Data Availability

The data underlying this article are available in the article and in its online supplementary resources, while further clarification can be provided upon request.

## Appendices

### Appendix 1: Summary of PIDA chapters

**Table:**

PIDA chapters	Content
1. Introduction	Introduces the Decision Aid and its purpose to enable women with IBD to make informed decisions about pregnancy. It is designed as a series of walk-through informational sections
2. Essential info	Sub-sections providing information about the impact of disease activity, nutrition, physical activity, and substance use on pregnancy outcomes in IBD
3. Tell us about you	A series of questions to allow patients to tailor the Decision Aid arms to their personal context
	(1) preconception or pregnancy
	(2) ulcerative colitis, Crohn’s disease, or unsure
	(3) prior surgeries (including options of J-pouch, colostomy/ileostomy, colectomy, ileal/ileocolonic resection) or no surgery
	(4) drop down options to generate a medication list
	(5) agree/disagree scale questions to establish the patient’s current opinions regarding the safety profile of their IBD medications during pregnancy, concern about adverse effects and intentions to continue medications before and during pregnancy
4. Preconception	Provision of information on the impact of IBD on fertility, inheritance of IBD, types of contraception and potential concerns in IBD. Available evidence for safety of the patient’s IBD medications in preconception is presented
5. Pregnancy	If the patient has listed prior surgery, the potential impact of the operation for pregnancy and delivery is described. Available evidence for safety of the patient’s IBD medications in pregnancy is presented
6. Postpartum	Provision of information on the impact of the patient’s IBD medications for infants including biologics, vaccinations, infection risk and breastfeeding. Information about post-partum medication use is described including pain management and timing of IBD medications after delivery
7. Summary	Opinion review with the agree/disagree questions from Sect. 3, followed by a printable summary of the patient’s Decision Aid

### Appendix 2: Patient Acceptability Questionnaire

LENGTH

My review of the decision aid took me ……. minutes.

On a scale of 1 to 5, I rate the length as:

**Table:**

1	2	3	4	5
Too short		Adequate		Excessive

READABILITY

On a scale of 1 to 5, I rate the ease to read as:

**Table:**

1	2	3	4	5
Too simplified		Appropriate		Too challenging

CONTENT AMOUNT

On a scale of 1 to 5, I rate the amount of information provided as:

**Table:**

1	2	3	4	5
Limited		Appropriate		Excessive

USEFULNESS (for me).

On a scale of 1 to 5, I rate how well the decision aid helped my understanding and decision making as:

**Table:**

1	2	3	4	5
More confusing		No difference		Useful

USEFULNESS (for others).

On a scale of 1 to 5, this is how I would recommend the decision aid to others in my situation as:

**Table:**

1	2	3	4	5
Not at all		Suggest		Highly Recommend

VALUES

On a scale of 1 to 5, I rate how well the decision aid helps me demonstrate what is important to me as:

**Table:**

1	2	3	4	5
Not at all		Adequate		Very Well

### Appendix 3: Clinician Acceptability Questionnaire

LENGTH

My review of the decision aid took me ……. minutes.

On a scale of 1 to 5, I rate the length as:

**Table:**

1	2	3	4	5
Too short		Adequate		Excessive

READABILITY

On a scale of 1 to 5, I rate the ease to read as:

**Table:**

1	2	3	4	5
Too simplified		Appropriate		Too challenging

CONTENT AMOUNT

On a scale of 1 to 5, I rate the amount of information provided as:

**Table:**

1	2	3	4	5
Limited		Appropriate		Excessive

USEFULNESS (for me).

On a scale of 1 to 5, I rate how well I expect the decision aid would help patient understanding and decision making as:

**Table:**

1	2	3	4	5
More confusing		No difference		Useful

USEFULNESS (for others).

On a scale of 1 to 5, this is how I would recommend the decision aid to patients:

**Table:**

1	2	3	4	5
Not at all		Suggest		Highly Recommend

VALUES

On a scale of 1 to 5, I rate how well the decision aid would likely help patients demonstrate what is important to them as:

**Table:**

1	2	3	4	5
Not at all		Adequate		Very Well

ACCURACY

Please list any concerns regarding the accuracy of data presented in the decision aid:
