# Supplementary material for: A Novel Decision Aid Improves Quality of Reproductive Decision-Making and Pregnancy Knowledge for Women with Inflammatory Bowel Disease
Source: Dig Dis Sci. 2022 Apr 30;67(9):4303–14. doi: 10.1007/s10620-022-07494-9 (PMC9352739; doi:10.1007/s10620-022-07494-9)
Supplement: Supplementary file 1 — Supplementary file1 (DOCX 34 KB) [file 10620_2022_7494_MOESM1_ESM.docx]

Supplementary Online Resources

**Article Title**: A Novel Decision Aid Improves Quality of Reproductive Decision-Making and Pregnancy Knowledge for Women with Inflammatory Bowel Disease

**Journal Name**: Digestive Diseases and Sciences

**Author Names**: Grace Wang*^1^, Neda Karimi*^2,3^, Laura Willmann^4^, Joseph Pipicella^3,4^, Joseph Descallar^2,3^, Katie O’Connor^5^, Luiza Peculis^6^, Yvette Leung^7^, Susan Connor^2,4^, Vivian Huang^1,5^, Astrid-Jane Williams^2,4^

**Corresponding Author**: Astrid-Jane Williams; astridjane.williams@health.nsw.gov.au

Online Resource 1. Number of analysed and missing responses for each outcome measure, according to reproductive status.

| **Outcome measure** | **Preconception (n=43)** | | **`** | |
| --- | --- | --- | --- | --- |
|  | Analysed | Missing | Analysed | Missing |
| **Decisional Conflict Scale** | n=41 | n=2 | n=33 | n=0 |
| **Preparation for Decision Making** | n=42 | n=1 | n=33 | n=0 |
| **Decision Self-Efficacy Scale** | n=41 | n=2 | n=33 | n=0 |
| **Crohn’s and Colitis Pregnancy Knowledge Score** | n=43 | n=0 | n=33 | n=0 |
| **Acceptability** | n=42 | n=1 | n=33 | n=0 |

Online Resource 2. Pre- and Post PIDA Decisional Conflict Scale scores including subscores by domain for preconception patients.

|  |  | **Pre-PIDA** | | **Post-PIDA** | | **Difference (Pre – Post)** | | | |  |  |  |  |
| --- | --- | --- | --- | --- | --- | --- | --- | --- | --- | --- | --- | --- | --- |
|  | **N** | **Mean** | **Standard Deviation** | **Mean** | **Standard Deviation** | **Mean** | **Lower 95% CI** | **Upper 95% CI** | **P-value** | **df** | **t** | **Effect size** | **Effect size 2** |
| **Total** | 41 | 38.95 | 21.31 | 26.77 | 16.79 | 12.18 | 7.08 | 17.27 | <0.0001 | 40 | 4.83 | 0.75 | 0.57 |
| **Uncertainty** | 41 | 45.12 | 27.00 | 35.37 | 24.06 | 9.76 | 3.10 | 16.41 | 0.0051 | 40 | 2.96 | 0.46 | 0.36 |
| **Informed** | 41 | 43.70 | 26.14 | 23.37 | 17.80 | 20.33 | 13.14 | 27.51 | <0.0001 | 40 | 5.72 | 0.89 | 0.78 |
| **Clarity** | 41 | 45.53 | 27.39 | 27.24 | 23.05 | 18.29 | 10.66 | 25.92 | <0.0001 | 40 | 4.85 | 0.76 | 0.67 |
| **Support** | 41 | 31.71 | 20.85 | 22.76 | 14.91 | 8.94 | 4.45 | 13.44 | 0.0002 | 40 | 4.02 | 0.63 | 0.43 |
| **Effective** | 41 | 31.25 | 18.70 | 25.46 | 17.08 | 5.79 | 1.03 | 10.55 | 0.0183 | 40 | 2.46 | 0.38 | 0.31 |

Online Resource 3. Pre- and Post PIDA Decisional Conflict Scale scores including subscores by domain for pregnant patients.

|  |  | **Pre-PIDA** | | **Post-PIDA** | | **Difference (Pre – Post)** | | | |  |  |  |  |
| --- | --- | --- | --- | --- | --- | --- | --- | --- | --- | --- | --- | --- | --- |
|  | **N** | **Mean** | **Standard Deviation** | **Mean** | **Standard Deviation** | **Mean** | **Lower 95% CI** | **Upper 95% CI** | **P-value** | **df** | **t** | **Effect size** | **Effect Size 2** |
| **Total** | 33 | 23.30 | 13.07 | 18.99 | 17.06 | 4.31 | 0.60 | 8.02 | 0.0242 | 32 | 2.37 | 0.41 | 0.33 |
| **Uncertainty** | 33 | 25.76 | 16.32 | 18.69 | 18.64 | 7.07 | 1.59 | 12.56 | 0.0131 | 32 | 2.63 | 0.46 | 0.43 |
| **Informed** | 33 | 28.54 | 17.68 | 21.46 | 22.54 | 7.07 | 0.71 | 13.43 | 0.0304 | 32 | 2.26 | 0.39 | 0.4 |
| **Clarity** | 33 | 25.00 | 16.80 | 19.95 | 21.94 | 5.05 | -1.08 | 11.18 | 0.1032 | 32 | 1.68 | 0.29 | 0.3 |
| **Support** | 33 | 18.43 | 14.55 | 17.42 | 17.23 | 1.01 | -3.65 | 5.67 | 0.6617 | 32 | 0.44 | 0.08 | 0.07 |
| **Effective** | 33 | 19.89 | 14.53 | 17.80 | 17.96 | 2.08 | -2.37 | 6.54 | 0.348 | 32 | 0.95 | 0.17 | 0.14 |

Online Resource 4. Nominal symmetry test results for pregnancy-related decisions in the context of IBD for preconception patients, pre- and post-review of PIDA.

|  | **Post-PIDA** | | | | | | | |
| --- | --- | --- | --- | --- | --- | --- | --- | --- |
|  | **1** | | **2** | | **3** | | **4** | |
| **Pre-PIDA** | **n** | **%** | **n** | **%** | **n** | **%** | **n** | **%** |
| **1** | 0 | 0 | 0 | 0 | 0 | 0 | 0 | 0 |
| **2** | 0 | 0 | 1 | 2.5 | 0 | 0 | 0 | 0 |
| **3** | 0 | 0 | 0 | 0 | 11 | 27.5 | **8*** | **20*** |
| **4** | 0 | 0 | 0 | 0 | 0 | 0 | 20 | 50 |
| 1. I am planning to not have children because of my IBD. 2. I am planning to not have children (unrelated to my IBD). 3. I would like to have children but I am afraid to do so because of my IBD. 4. I would like to have children and I have no major concern regarding my IBD.   *Significant (*p*=0.00781) | | | | | | | | |

Online Resource 5. Nominal symmetry test results for pregnancy-related decisions in the context of IBD for pregnant patients, pre- and post-review of PIDA.

|  | **Post-PIDA** | | | | | | | | | |
| --- | --- | --- | --- | --- | --- | --- | --- | --- | --- | --- |
|  | **1** | | **2** | | **3** | | **4** | | **5** | |
| **Pre-PIDA** | **n** | **%** | **n** | **%** | **n** | **%** | **n** | **%** | **n** | **%** |
| **1** | 17 | 51.52 | 0 | 0 | 0 | 0 | 0 | 0 | 0 | 0 |
| **2** | 4 | 12.12 | 4 | 12.12 | 1 | 3.03 | 0 | 0 | 0 | 0 |
| **3** | 0 | 0 | 0 | 0 | 1 | 3.03 | 0 | 0 | 0 | 0 |
| **4** | 1 | 3.03 | 0 | 0 | 0 | 0 | 3 | 9.09 | 0 | 0 |
| **5** | 0 | 0 | 1 | 3.03 | 0 | 0 | 1 | 3.03 | 0 | 0 |
| 1. I am planning to continue my current IBD medications throughout all of my pregnancy. 2. I am planning to continue my current IBD medications through to third trimester. 3. I am planning to stop my current IBD medications now. 4. I am planning on continuing not being on any IBD medication throughout all of my pregnancy. 5. I am planning on starting IBD medications during my pregnancy. | | | | | | | | | | |

Online Resource 6. Qualitative review of PIDA provided by clinician participants, divided by themes.

| **Main themes** | **Sub-themes** |
| --- | --- |
| Accuracy | - Preterm birth rate - Diet and food storage during pregnancy - Biologic transfer to placenta - Risk of IBD - Medication and timing of recommencement after delivery |
| Language | - Presentation of complex and detailed medical concepts without adequate background information (e.g., discussion surrounding laboratory values in pregnancy) - Use of Australian and Canadian terms - Communication of risk |
| Technical issues | - DA algorithm (e.g., differentiation between considering and actually having a surgery; timing of information provision regarding vaccination) - Broken link |
| Effect | - Caution with provision of too much information, especially that which is based on case reports, as this may deter medication adherence |
| Content | Suggestions to provide:   - Further safety netting information - A reference to the drug and lactation database - Information on folic acid supplements, risk of smoking in pregnancy, risk of infection in infants, risk of miscarriage/birth defects in the healthy population, IBD medications, and infant vaccinations |
| Visual representation | Use of visual representation including:   - A visual analogue scale for medication intake behavior - Color-coding - Interval plots for conveying risk |
